# Puerperal Infection in the Male

**Published:** 1882-01

**Authors:** 


### PUERPERAL INFECTION IN THE MALE.

During the prevalence of a severe epidemic of puerperal fever in Pollenza a woman in childbed was attacked by a fever occurring in paroxysms and resembling intermittent fever. A few days after the last paroxysm, when the patient felt perfectly well, her husband attempted to have sexual intercourse with her, but the onset of intense pain in the region of the frenulum compelled him shortly to desist. He stated that he was sure something must have been torn at the time, but he had not noticed any bleeding. In a short while the pain subsided. Twenty-four hours later, however, a chill occurred, followed by intense fever, with remission of all the symptoms on the following morning. In the evening the chill and fever recurred. On the third day, the right inguinal glands became swollen. When called in to see the patient on the fourth day, Lapponi diagnosed erysipelas of the skin of the penis, lymphangitis, and lymphadenitis. The erysipelatous inflammation continued to spread, the skin becoming gangrenous at several points. On the sixth day the patient died, with well marked symptoms of septicæmia. Although not able to discover any laceration at the time of the first examination. Lapponi still assumes that the point of infection was a slight tear of the frenulum, which the patient suffered during the unsuccessful attempt at coition.—*Centralbatt fuer Gynækalogie*, *Medical Record.*

